# Supplementary material for: A spatially-resolved transcriptional atlas of the murine dorsal pons at single-cell resolution
Source: Nat Commun. 2024 Mar 4;15:1966. doi: 10.1038/s41467-024-45907-7 (PMC10912765; doi:10.1038/s41467-024-45907-7)
Supplement: Supplementary file 1 — Supplementary Information [file 41467_2024_45907_MOESM1_ESM.pdf]

SUPPLEMENTARY FIGURES AND LEGENDS

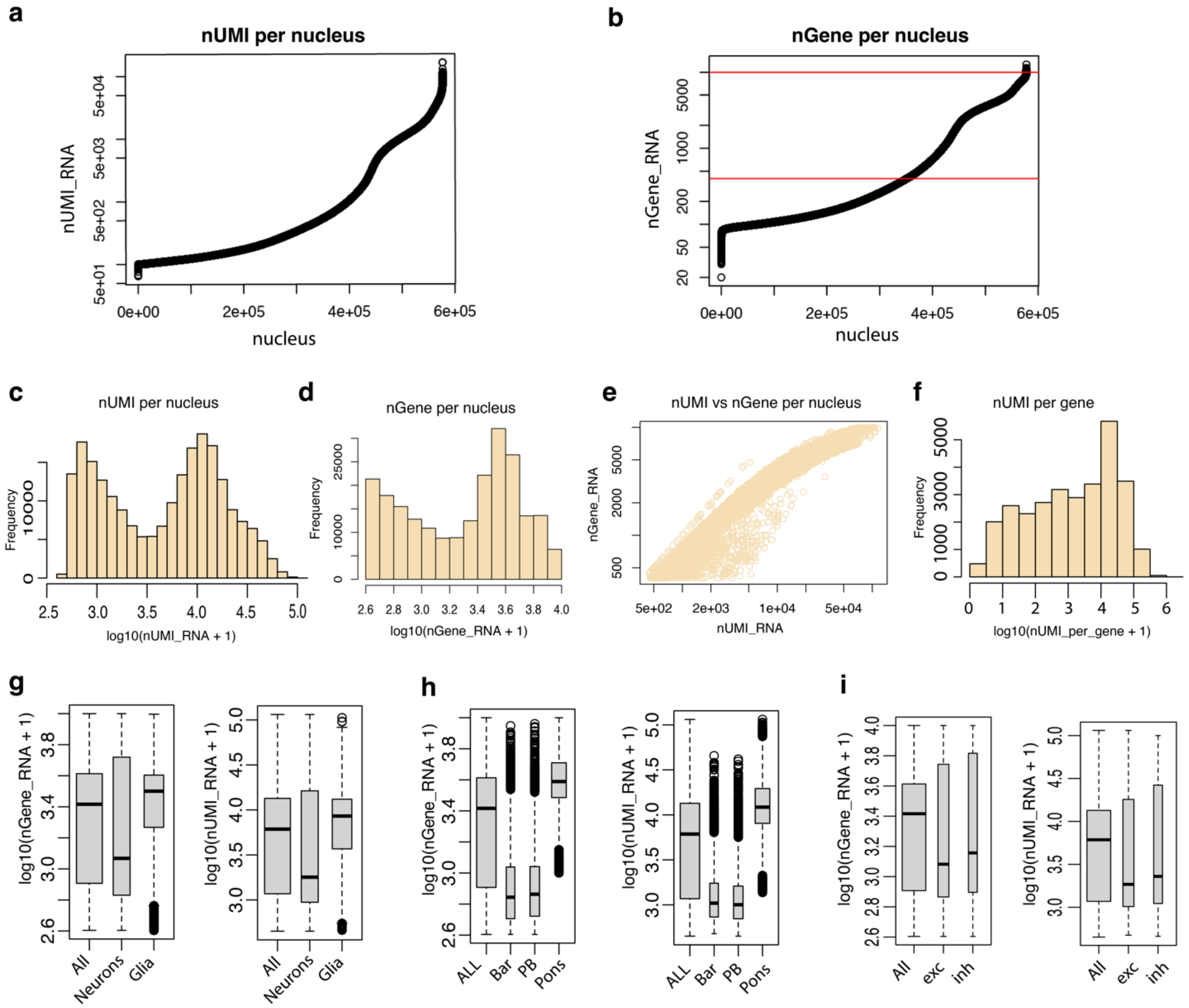

Supplementary Figure 1

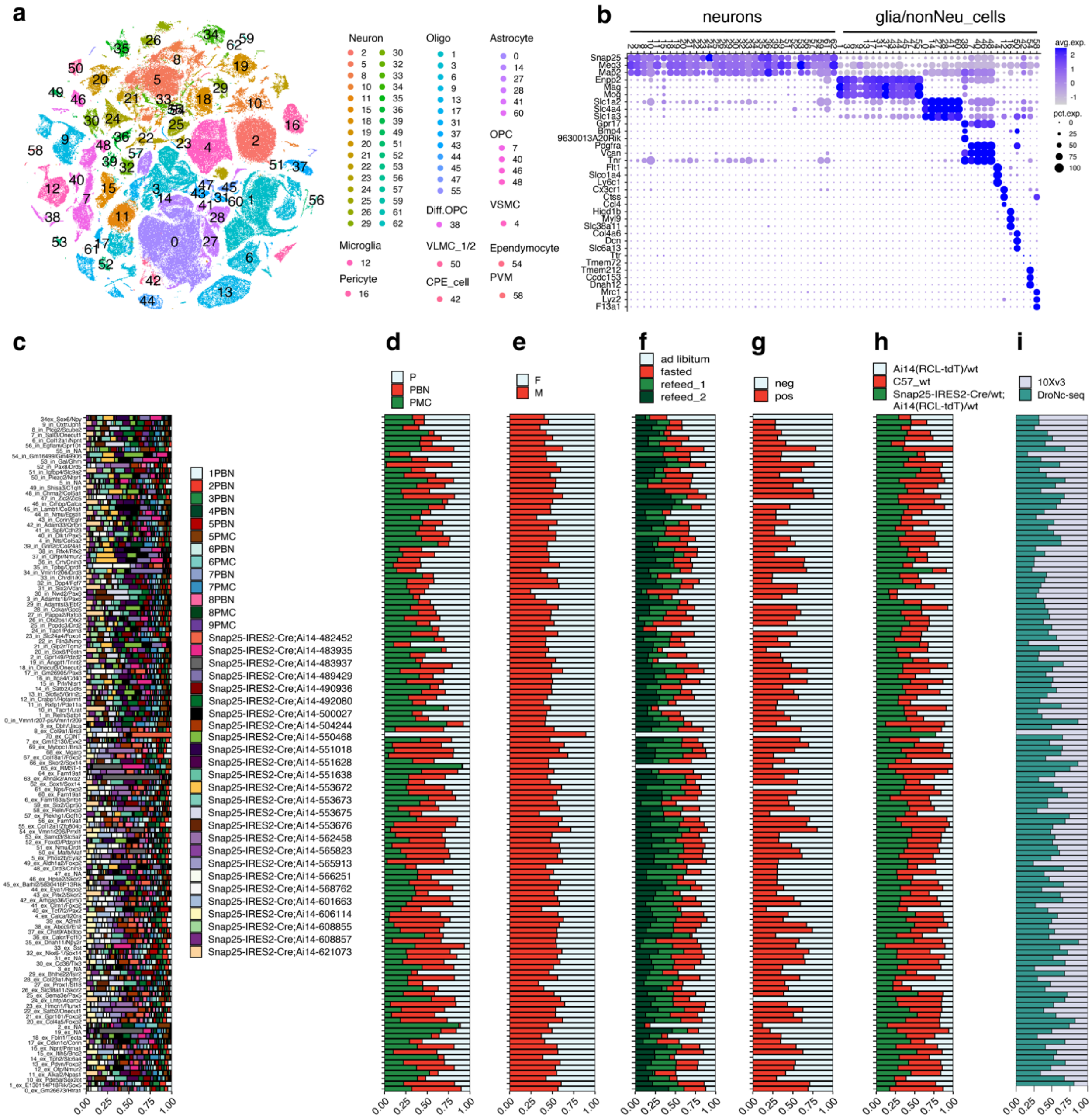

Supplementary Figure 2

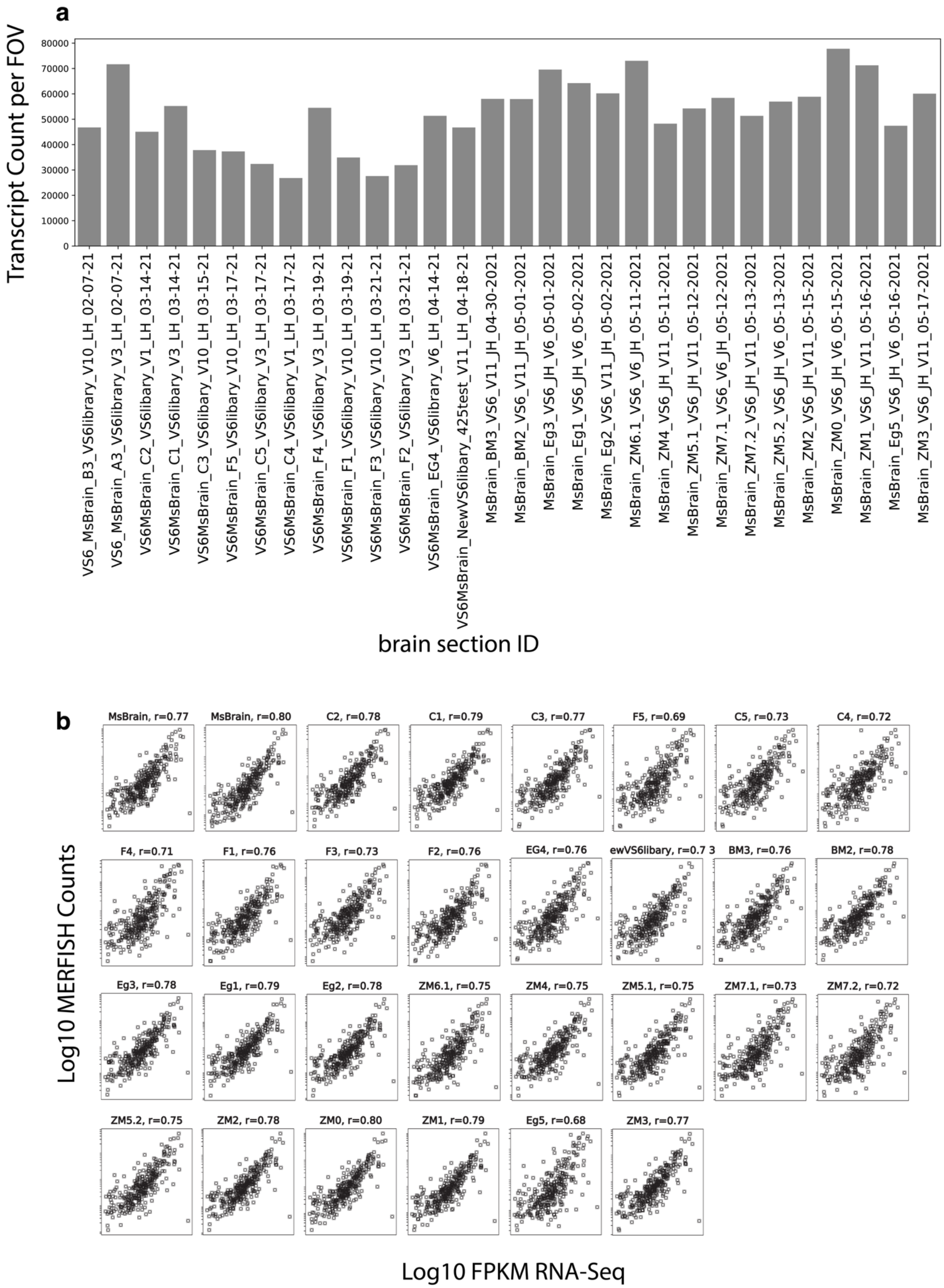

Supplementary Figure 3

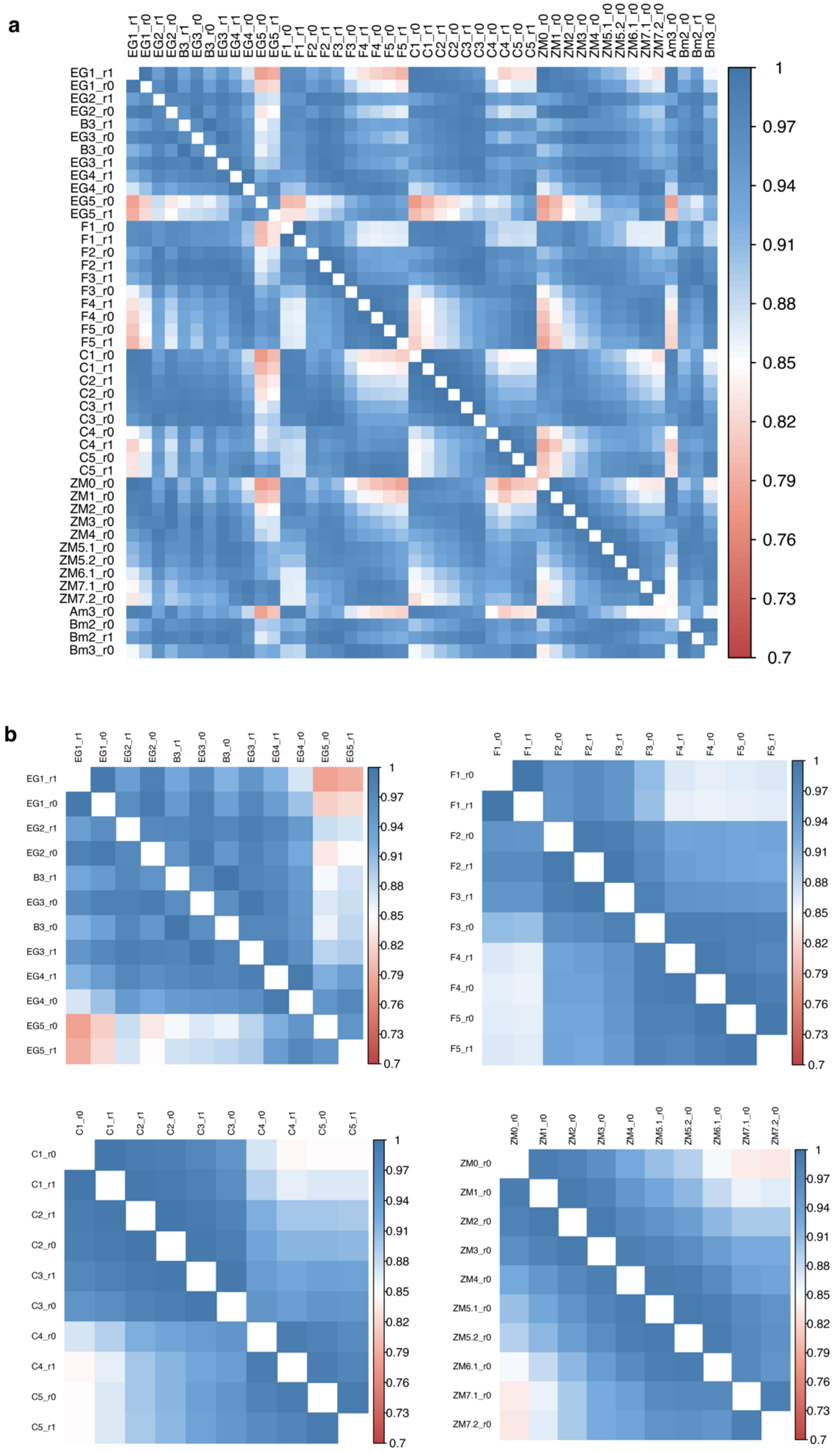

9 **Supplementary Figure 4**

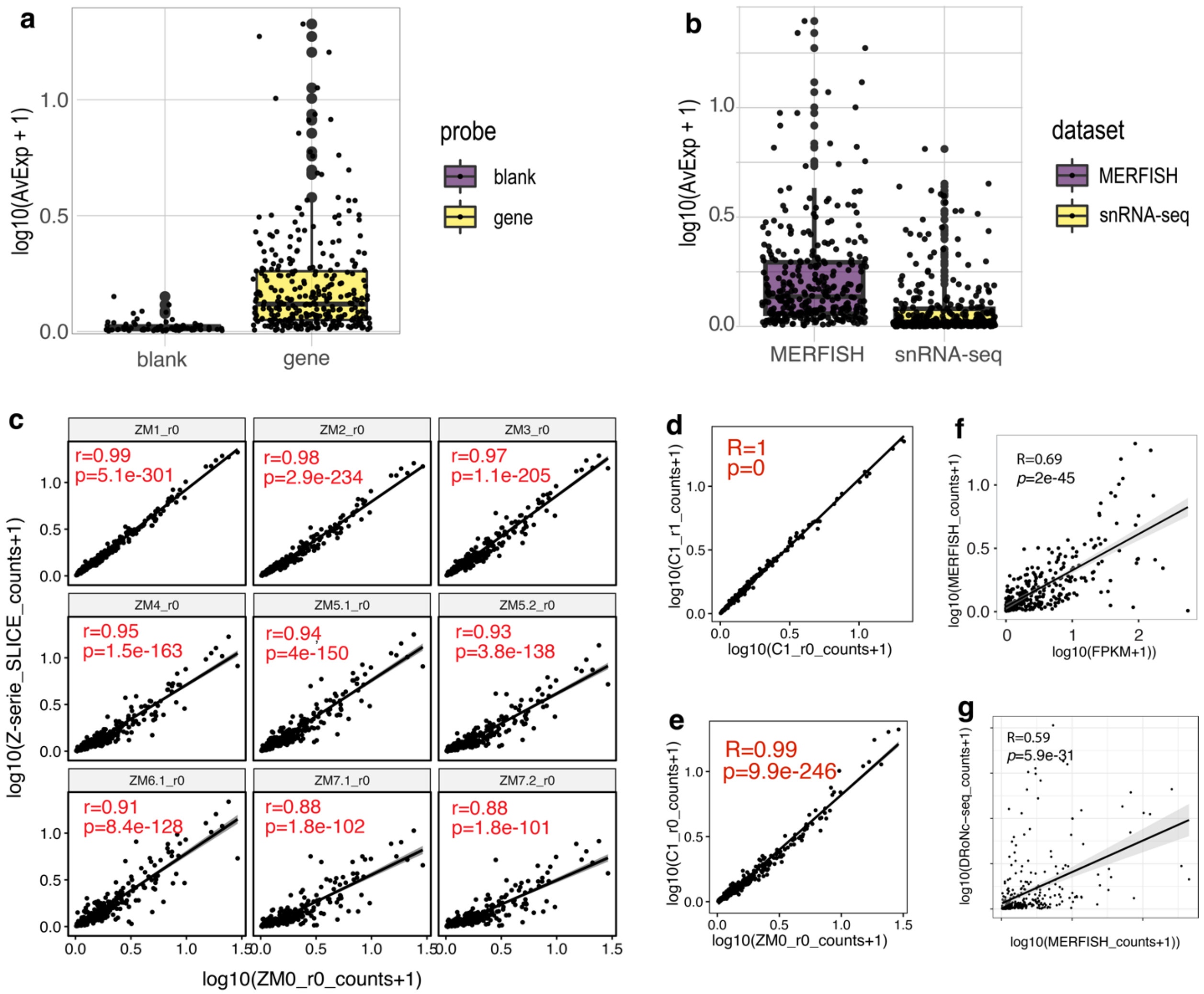

10 **Supplementary Figure 5**

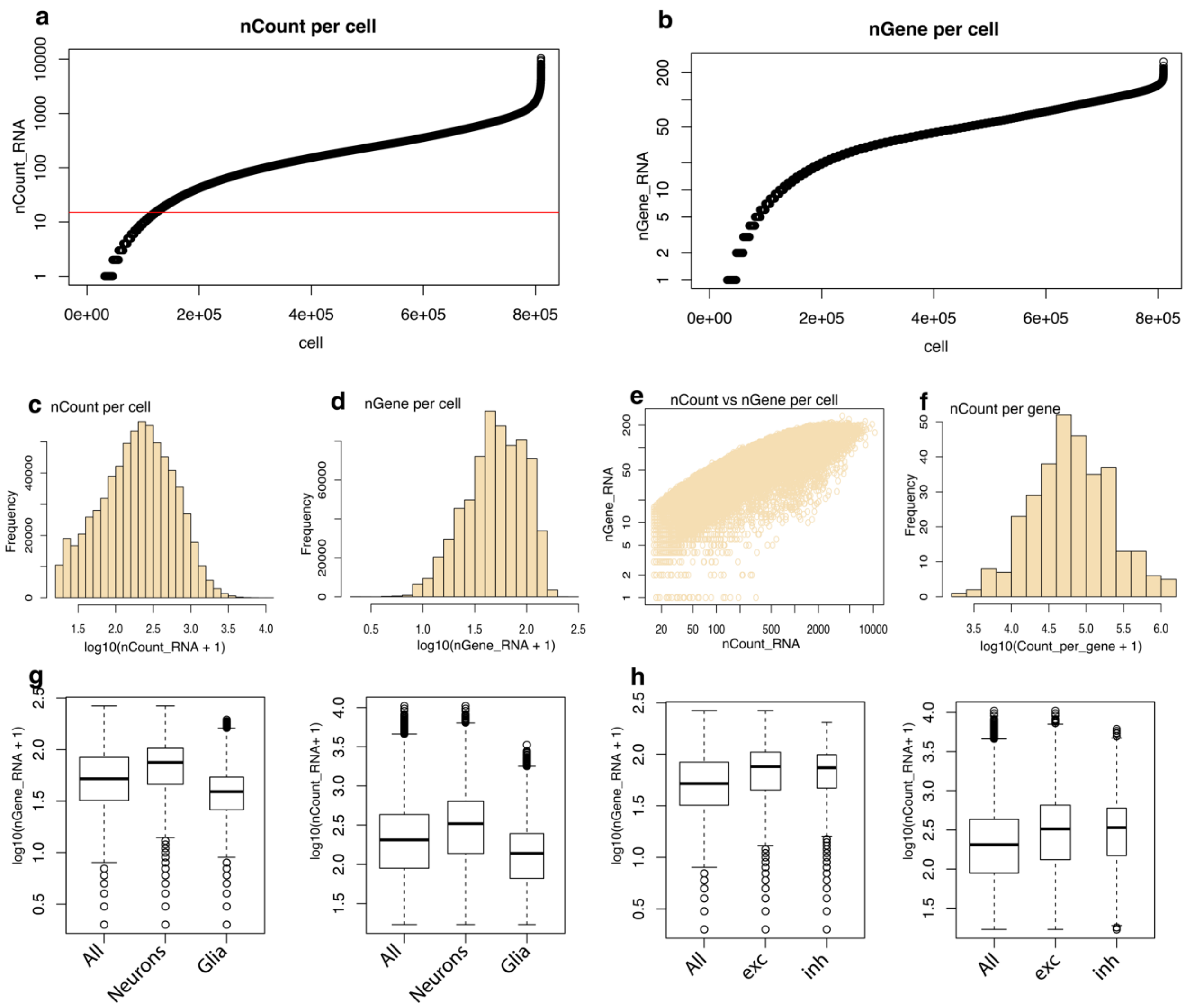

11 **Supplementary Figure 6**

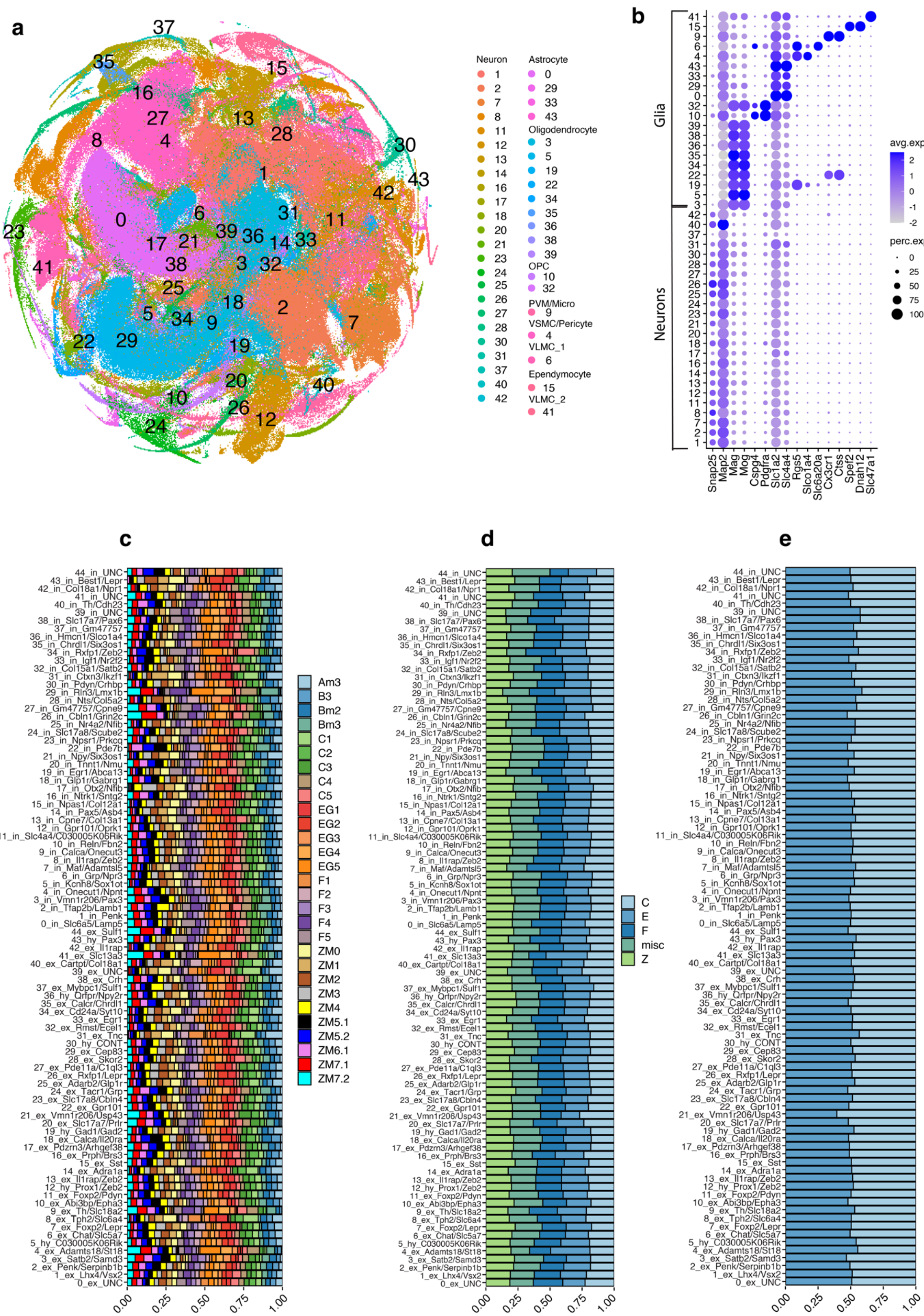

Supplementary Figure 7

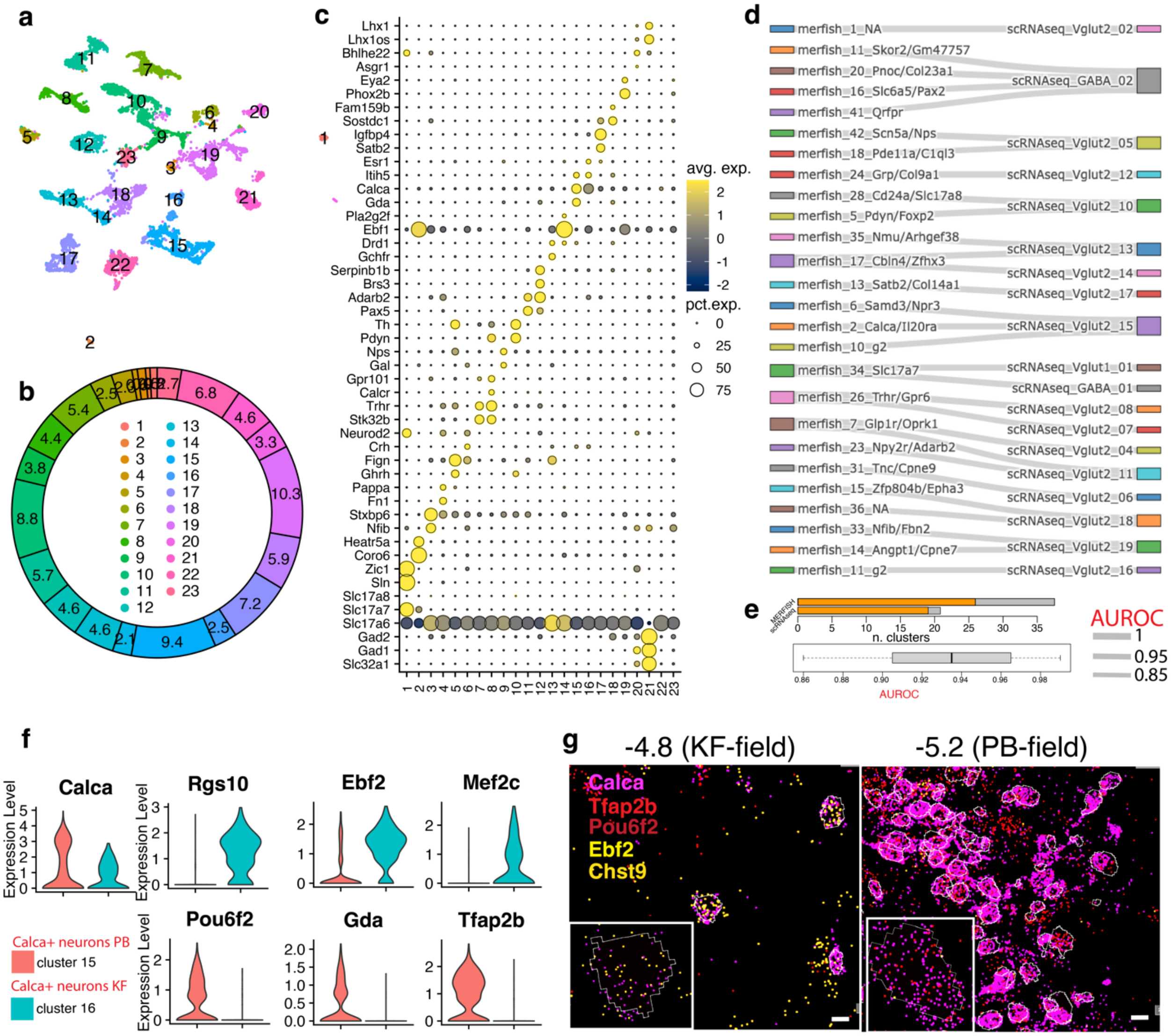

Supplementary Figure 8

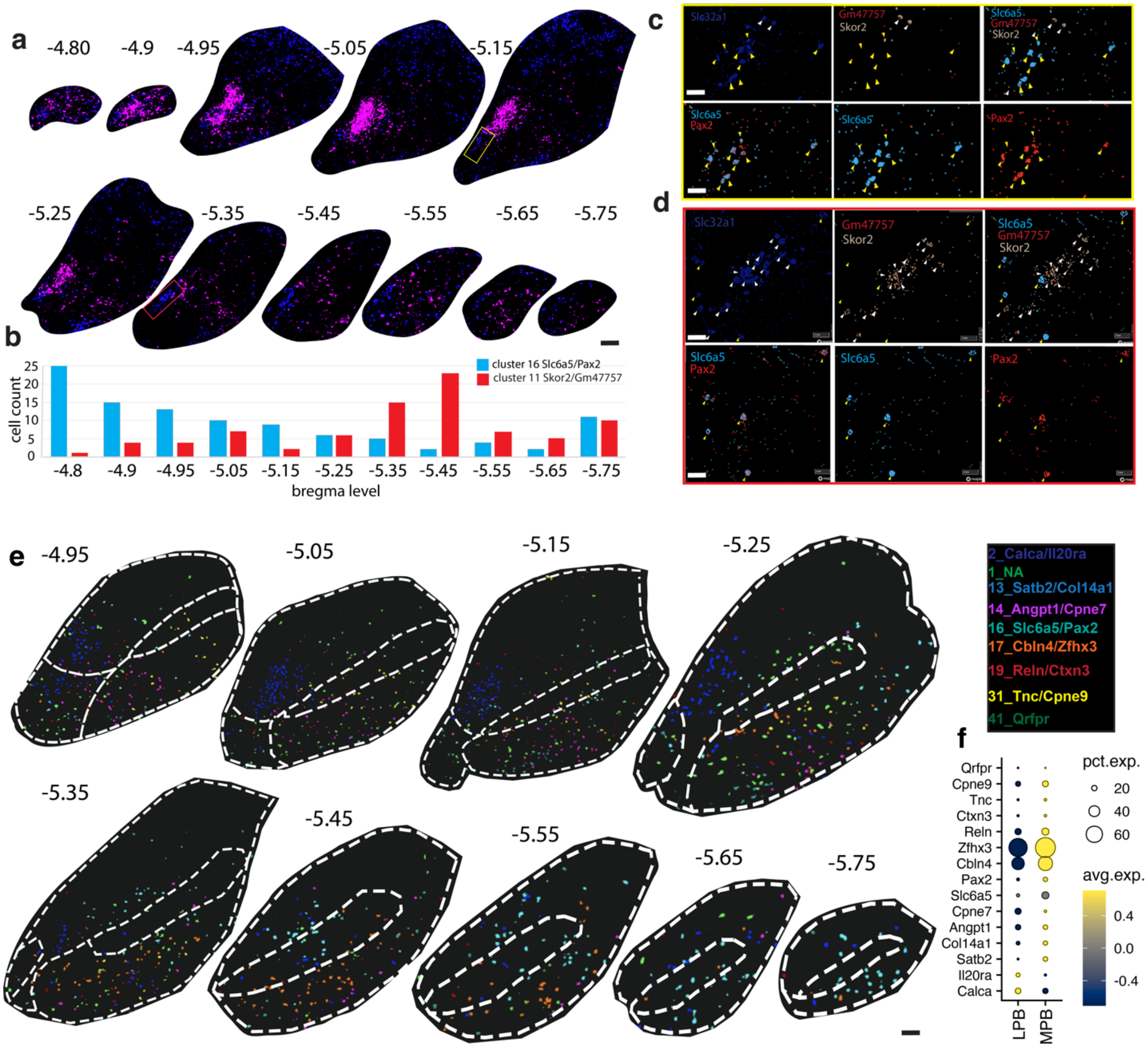

Supplementary Figure 9

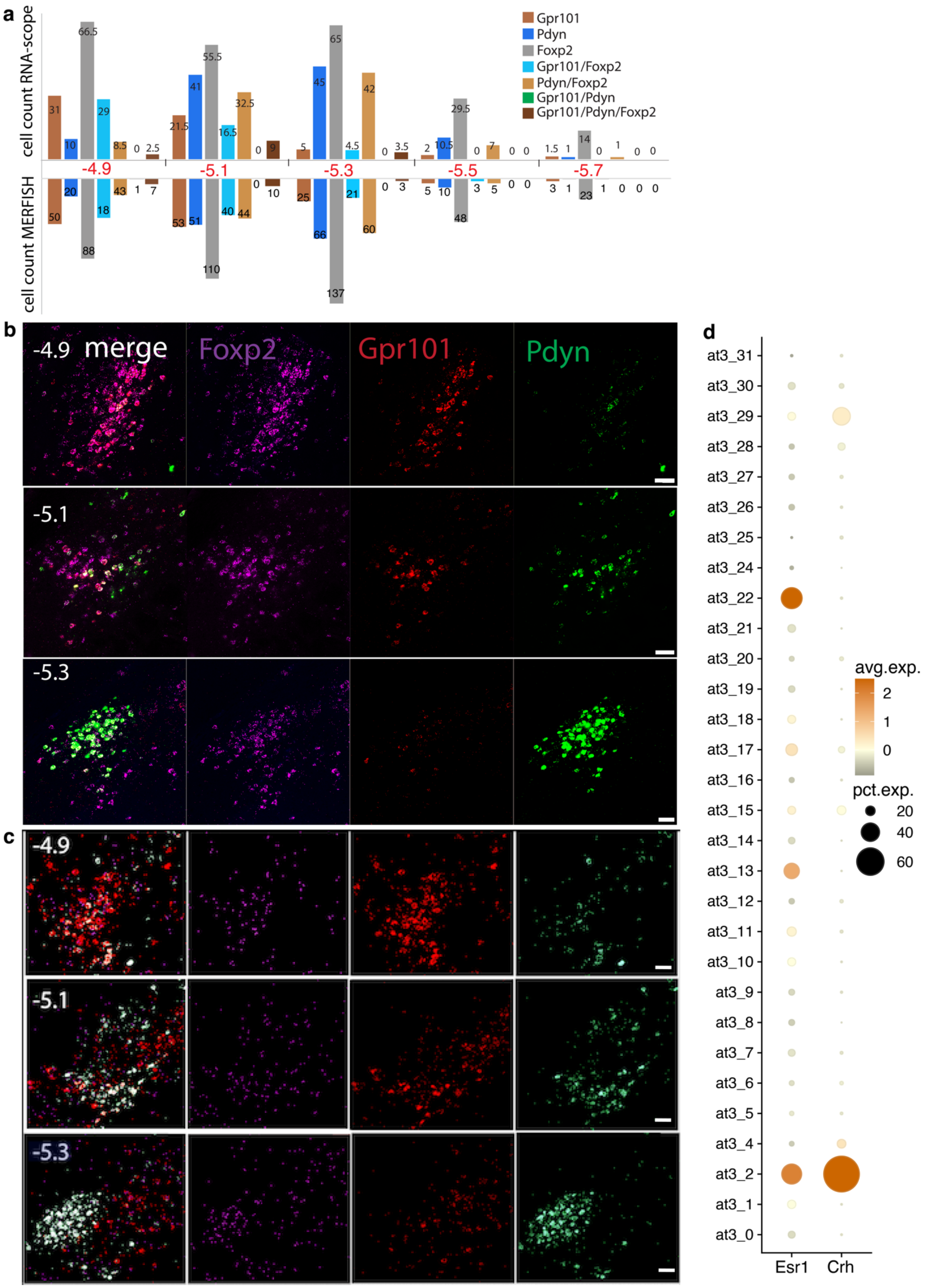

Supplementary Figure 10

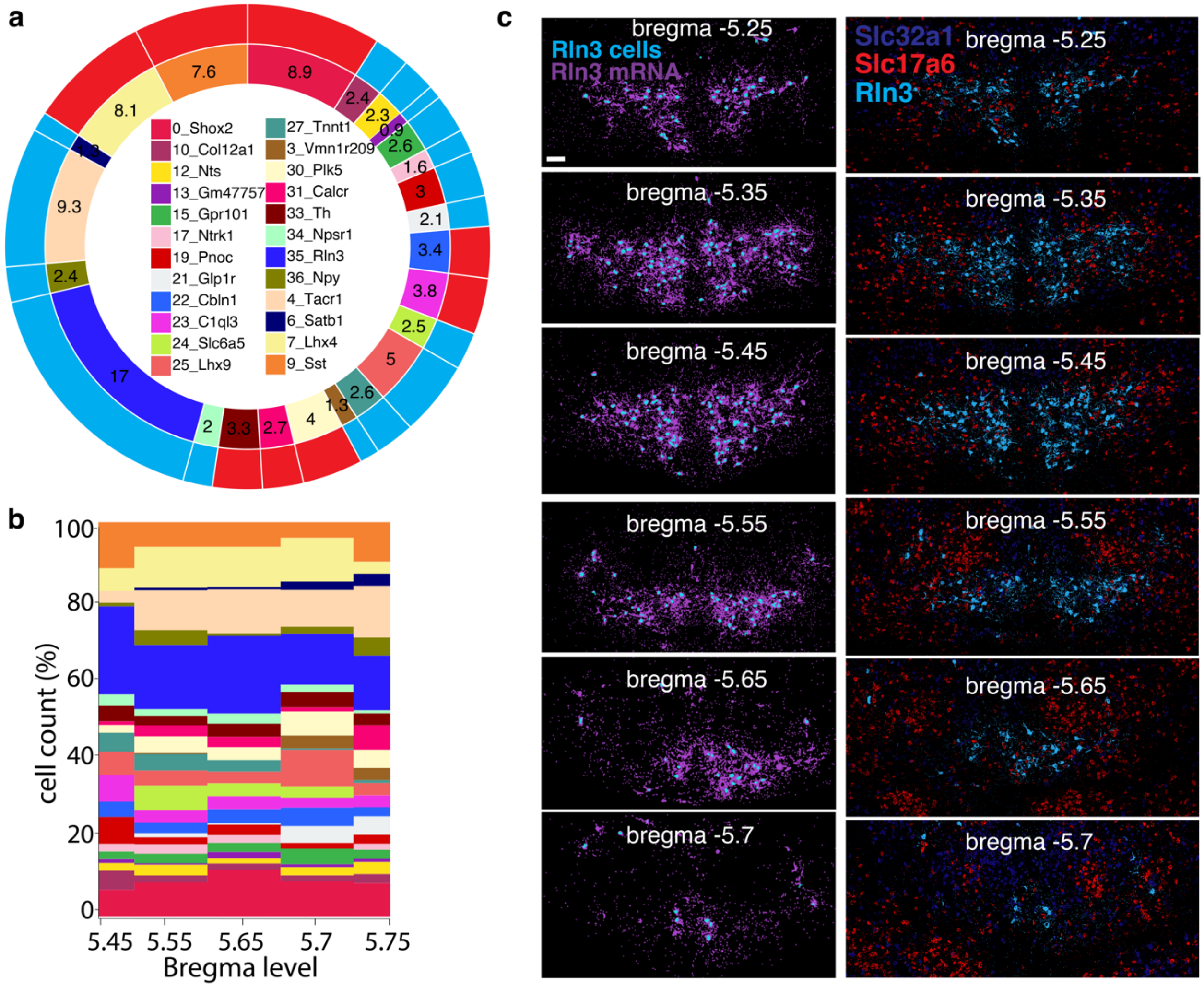

Supplementary Figure 11

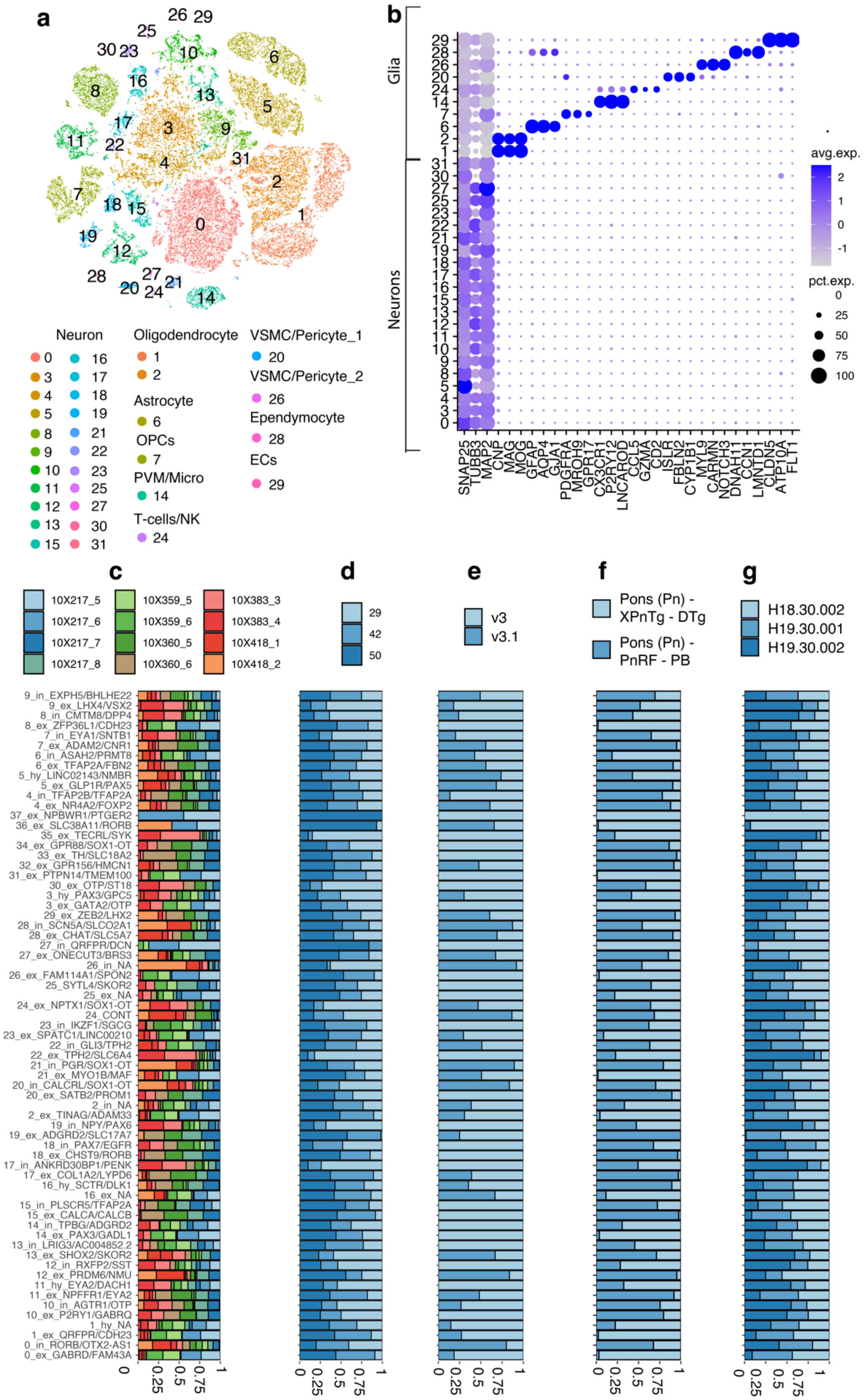

Supplementary Figure 12

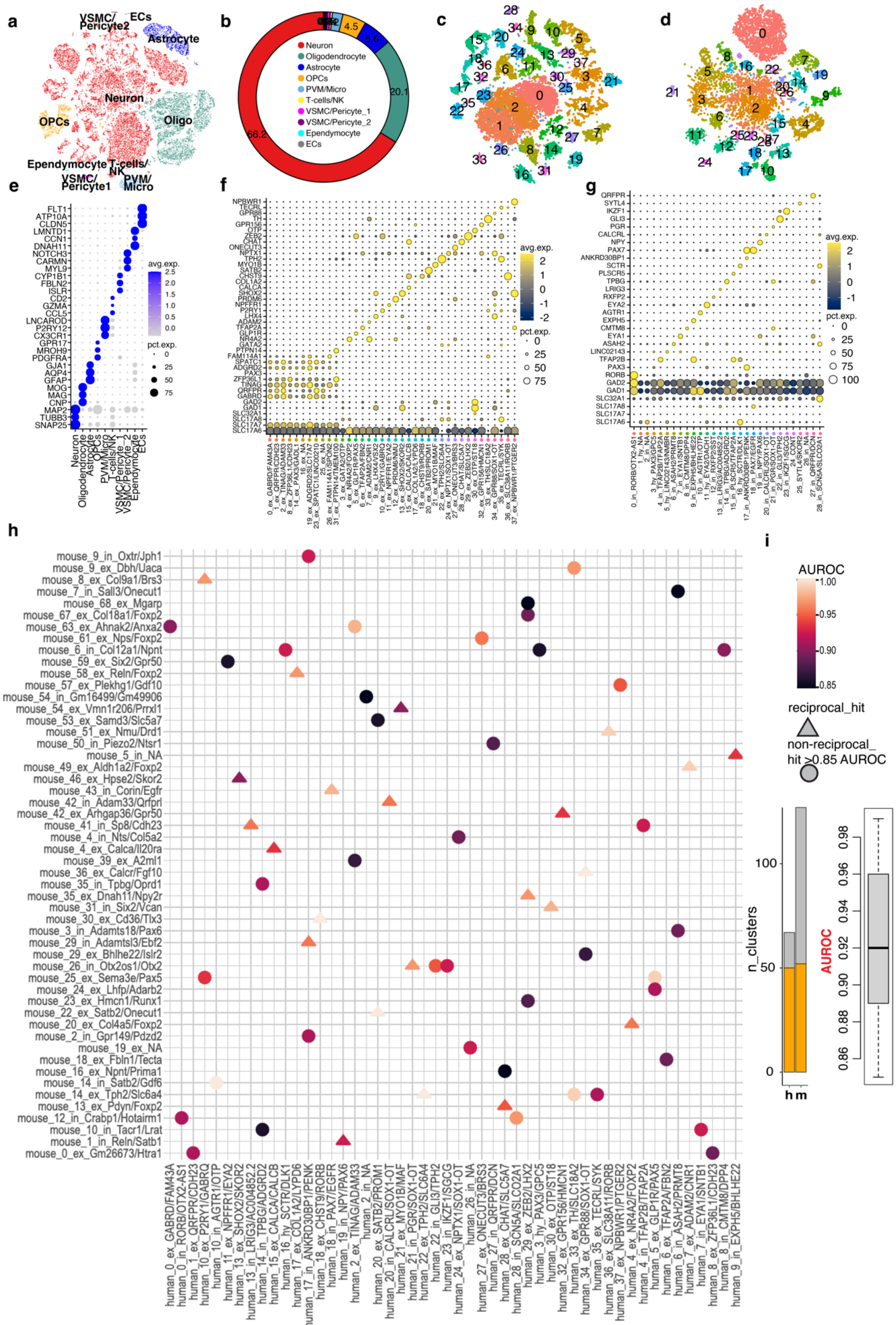

23 **Supplementary Figure 13**

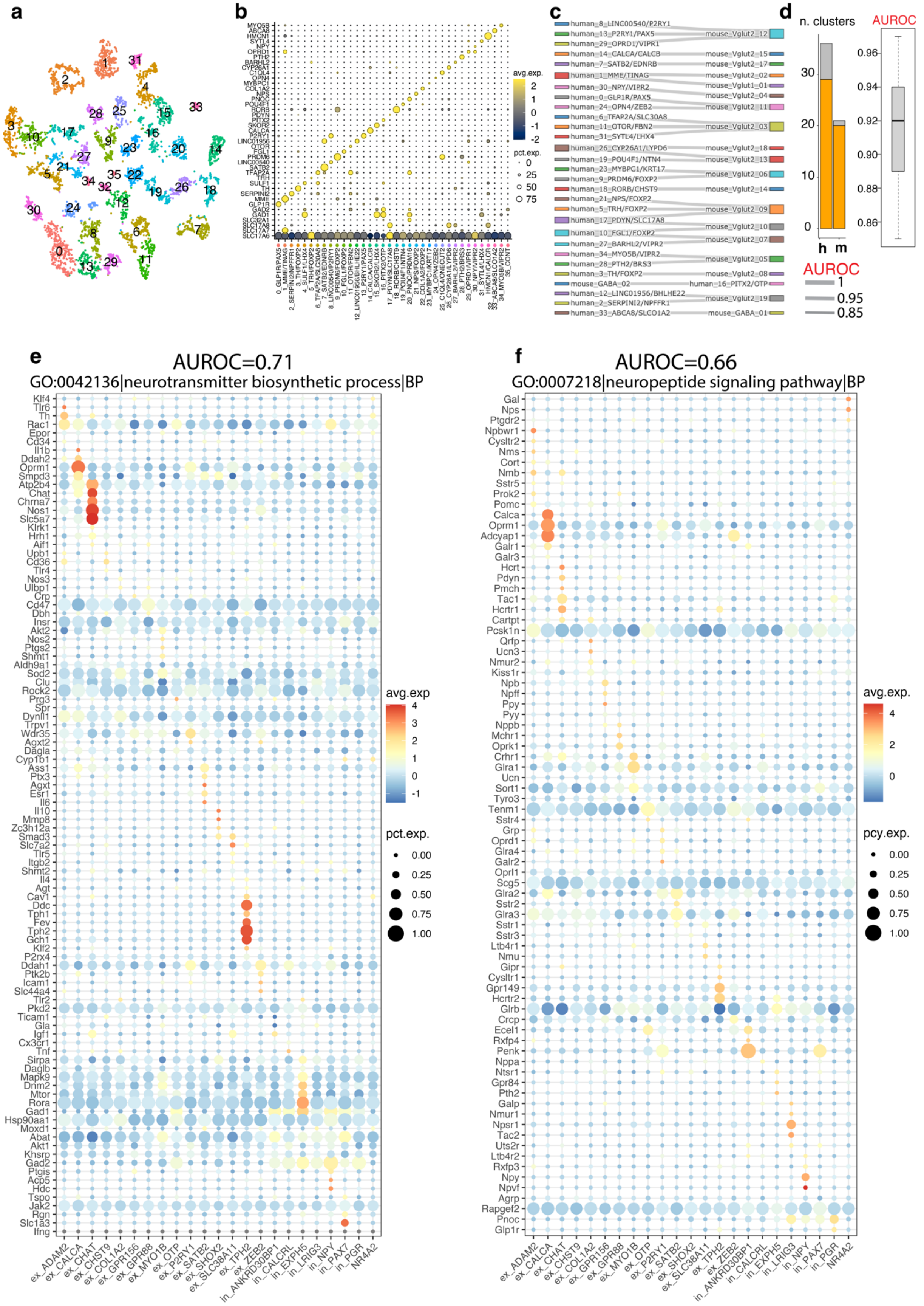

 Supplementary Figure 14

Supplementary Figure 1. Data distribution and QC plots of snRNA-seq dataset a-b) Scatter plots showing the distribution, plotted in crescent order, of nUMI (**a**) and nGene (**b**) for all nuclei in the snRNA-seq dataset before filtering was applied. The two red lines indicate the thresholds set to filter out potential empty/ low-quality droplets (nGene <400) or doublets (nGene >10000). **c**) Bar plot of nUMI per nucleus. **d**) Bar plot of nGene per nucleus. **e**) Scatter plot of nUMI versus nGene. **f**) Bar plot of nUMI per gene. **g**) Box plot displaying the distribution of nGene (left) and nUMI (right) in "all nuclei", "neurons", "and glia/ non-neuronal" cells. **h**) Box plot displaying the distribution of nGene (left) and nUMI (right) in all nuclei of the "merged", "PB", "Bar", and "Pons" datasets. **i**) Box plot displaying the distribution of nGene (left) and nUMI (right) in "all nuclei", "excitatory", and "inhibitory" neurons. The black middle line denotes the median value (50th percentile), while the grey box contains the 25th to 75th percentiles of the dataset. The black whiskers mark the 5th and 95th percentiles, and values beyond these upper and lower bounds, marked with black dots, are considered outliers. Bar, Barrington's nucleus; PB, Parabrachial Nucleus; "exc", excitatory neurons; "inh" inhibitory neurons; "All" all nuclei; nUMIs, number of unique molecular identifiers; nGene, number of unique gene counts.

Supplementary Figure 2. Seurat clustering of the dPnTg nuclei profiled by snRNA-seq. a) t-SNE plot of 222,592 nuclei color-coded by annotated cell cluster. The legend on the right assigns each cluster a color and a cell type. **b**) Dot plot of 35 marker genes used to distinguish all main cell types belonging to "glia/non-neuronal cells" and "neurons" groups. 3 markers were plotted for all cell types except for CPE cells, where only two markers were used. **c-i**) Bar plot representing the contribution, expressed as % of nuclei (x-axis), of each experimental batch (c), dissection strategy (d), gender (e), feeding regime (f), CO2 treatment (g), mouse strain (h), and snRNA-seq technology (i) to each neuronal cluster (y-axis). All differentially expressed genes in the dot plot have an average log fold-change >0.25 and an adjusted p-value <0.01. Test used: *Wilcoxon Rank Sum two-sided Bonferroni-corrected Test*. Bar, Barrington's nucleus; PB, parabrachial nucleus; M, male; F, female; OPC, oligodendrocyte precursor cells; PVM, perivascular macrophages; VSMC, vascular smooth muscle cells; VLMCs, vascular and leptomeningeal cells; CPE, choroid plexus epithelial cells; t-SNE, t-distributed stochastic neighbor embedding; Diff.OPC, immature oligodendrocytes; NA, no marker detected; CONT, glia contamination.

Supplementary Figure 3. Transcript count per FOV and correlation between the average gene expression of 315 genes in MERFISH versus mouse brain bulk RNA-seq datasets. a) Bar plot showing the total Transcript Count per FOV (y-axis) across each MERSCOPE slide (x-axis) of the 315 MERFISH-assessed genes. **b**) Scatter plots representing the Pearson's *r* correlation coefficient of the average gene expression of 315 MERFISH-assessed genes between the whole mouse brain bulk RNA-seq and each MERSCOPE slide. For whole brain bulk RNA-seq, the average expression is reported as log10 FPKM, whereas for MERFISH, as log10 raw counts. All bar and scatter plots in panels a and b, respectively, except those representing BM3, Am3, and Z-series, refer to 2 coronal sections placed on the same slide and processed together. FOV, field of view; FPKM, Fragments per kilobase of transcript per million mapped fragments

Supplementary Figure 4. Correlation plots of the average expression of 315 genes between MERFISH sections. **a**) Correlation plot of the average expression of 315 genes among 46 MERFISH sections. **b**) Correlation plots of the average expression of 315 genes among 42 MERFISH sections grouped in 4 series of 10 sequential sections (except E-series, which includes 12 sections). Each series represents an experimental batch (i.e., a mouse). For all plots, a color-coded legend indicates Pearson's *r* correlation coefficient between two

sections. The white diagonal represents the correlation between identic sections corresponding to 1.

Supplementary Figure 5. Specificity, sensitivity, and reproducibility of MERFISH assay. a) Box plot of the average expression level of 315 MERFISH-assessed genes (specific signal) versus 60 scrambled probes (non-specific signal). **b**) Box plot displaying the average expression level of 315 genes assessed by snRNA-seq versus MERFISH technology**.** The black middle line denotes the median value (50th percentile), while the box contains the 25th to 75th percentiles of the dataset. The black whiskers mark the 5th and 95th percentiles, and values beyond these upper and lower bounds, marked with black dots, are considered outliers. **c**) Scatter plots representing the Pearson's *r* correlation coefficient calculated on the average expression of 315 genes between the section ZM0_r0 and the other nine sequential sections of the Z-series. **d-e**) Scatter plots representing the Pearson's *r* correlation coefficient calculated on the average expression of 315 genes between two sequential sections of the C-series (d) and two sections from two different series representing the same bregma level (e). **f-g**) Scatter plots representing Spearman's rho correlation coefficient of the average expression of 315 genes in MERFISH versus bulk RNA-seq from the whole mouse brain (f) and snRNA-seq from this study (g).

Supplementary Figure 6. Data distribution and QC plots of MERFISH dataset a-b) Scatter plots showing the distribution, plotted in crescent order, of nCount (a) and nGene (b) for all cells in the MERFISH dataset before filtering was applied. The red line corresponds to the threshold to eliminate potential low-quality cells or segmentation artifacts (nCount >15). **c**) Bar plot of nCount per cell. **d**) Bar plot of nGene per cell. **e**) Scatter plot of nCount versus nGene per cell. **f**) Bar plot of nCount per gene. **g**) Box plot of the distribution of nGene (left) and nCount (right) in "all cells", "neurons", and "glia/non-neuronal cells". **h**) Box plots of the distribution of nGene (left) and nCount (right) in "all cells", "excitatory", and "inhibitory" neurons. The black middle line denotes the median value (50th percentile), while the grey box contains the 25th to 75th percentiles of the dataset. The black whiskers mark the 5th and 95th percentiles, and values beyond these upper and lower bounds, marked with black dots, are considered outliers. "exc", excitatory; "inh", inhibitory; "All" all cells; nCount, number of raw counts; nGene, number of unique gene counts.

Supplementary Figure 7. Seurat clustering of all dPnTg cells profiled by MERFISH. a) t-SNE plot of 685,289 cells color-coded by annotated cell cluster. **b**) Dot plot of 16 cell markers used to differentiate all main cell types belonging to "neurons" and "glia/non-neuronal cells" classes. For all cell types, two markers were plotted except for VLMC type I and type II, where only one marker was plotted. **c-e**) Bar plot representing the contribution, expressed as % of cells (x-axis), of each experimental batch (c), series (d), and gender (e) to each neuronal cluster (y-axis). All batches, except the section Am3 and those from the Z series, refer to two coronal sections on a slide processed together. A series represents a mouse. All differentially expressed genes in the dot plot have an average log fold-change >0.25 and an adjusted p-value <0.01. Test used: *Wilcoxon Rank Sum two-sided Bonferroni-corrected Test*. t-SNE, t-distributed Stochastic Neighbor Embedding; OPC, oligodendrocyte progenitor cell; PVM/Micro, perivascular macrophages/ microglia; VSMC, vascular smooth muscle cells; VLMC, vascular and leptomeningeal cell; misc, miscellaneous; M, male; F, female; NA, no marker detected; CONT, glia contamination.

Supplementary Figure 8. Seurat clustering of PB/KF neurons from Pauli et al. dataset and correspondence with MERFISH atlas 2 (PB atlas). a) UMAP plot of 8,439 putative PB/KF neurons whose transcriptome was profiled by scRNA-seq. **b**) Donut plot depicting the fraction (%) of each neuronal cluster /total neurons. Color-coded legend is in the middle of the plot. **c**)

Dot plot of the expression level of each neuronal cluster's top 2 marker genes. **d**) Sankey plot depicting the cluster correspondence between MERFISH atlas 2 (PB) and scRNA-seq dataset from Pauli et al. The thickness of the line reflects the AUROC score. **e**) Top: stacked bar plot showing the number of clusters with a match (orange) over the total clusters (grey) identified by MERFISH and scRNA-seq approaches. Bottom: boxplot showing the AUROC scores distribution. The black middle line denotes the median value (50th percentile), while the grey box contains the 25th to 75th percentiles of the dataset. The black whiskers mark the 5th and 95th percentiles, and values beyond these upper and lower bounds, marked with black dots, are considered outliers. **f**) Violin plots showing the expression level of 7 genes in cluster 15 (PB) versus 16 (KF) of the Pauli et al. dataset. **g**) MERFISH images showing the expression level of 5 genes at -4.8 (KF-field) and -5.2 (PB-field) bregma levels. (scale bar: 25 µm). All differentially expressed genes in the dot plot have an average log fold-change >0.25 and an adjusted p-value <0.01. Test used: *Wilcoxon Rank Sum two-sided Bonferroni-corrected Test*. UMAP, Uniform Manifold Approximation and Projection; AUROC, area under the receiver operator characteristic curve; NA, no marker detected; CONT, glia contamination.

Supplementary Figure 9. In-depth characterization of the MPB. a) MERFISH images showing the distribution of *Calca* and *Slc32a1* across the entire PB complex. *Calca* is in magenta, and *Slc32a1* is in blue. (scale bar: 200 µm). **b**) Bar plot of the frequency distribution of clusters at2_11 (red) and at2_16 (cyan) indicated as absolute cell count (y-axis) across bregma levels from -4.8 to -5.75 (x-axis). **c-d**) Representative images of two sampled areas from panel (a) highlighted in yellow (c) and red(d) where clusters at2_16 (yellow arrows) and at2_11 (white arrows) are prevalent on their counterpart, respectively. (scale bar: 75 µm). Quantification and images of all levels are reported in Supplementary Table 14. **e**) MERFISH image showing only clusters residing in the MPB and cluster at2_2 in the LPBE as reference. Legend is in the top right corner. (scale bar: 175 µm). **f**) Dot plot depicting the average expression level of the top marker for the MPB clusters and the cluster at2_2 as control. All differentially expressed genes in the dot plot have an average log fold-change >0.25 and an adjusted p-value <0.01. Test used: *Wilcoxon Rank Sum two-sided Bonferroni-corrected Test*. Source Data are provided as Source Data file.

Supplementary Figure 10. MERFISH-detected clusters, at2_5 (*Foxp2+*/*Pdyn+*) and at2_26 (*Foxp2+*/*Gpr101+*), confirmed by *in situ* RNA-scope. a) Bar plots depicting the cell counts (y-axis) of 7 gene combinations (as per legend) relative to panels (b) and (c) across 3 sequential levels (x-axis). Quantification and images of all levels, including -those not reported in panels (b) and (c), are in Supplementary Table 16-17. **b-c**) RNA-scope (b) and MERFISH (c) assays probing *Foxp2*, *Pdyn,* and *Gpr101* transcripts at bregma levels -4.9, -5.1, and -5.3 are shown as single channels and overlay images. They are quantified in panel a. (scale bar: 50 µm). **d**) Dot plot of *Esr1* and *Crh* gene expression in MERFISH atlas 3. All differentially expressed genes in the dot plot have an average log fold-change >0.25 and an adjusted p-value <0.01. Test used: *Wilcoxon Rank Sum two-sided Bonferroni-corrected Test*. Source Data are provided as Source Data file.

Supplementary Figure 11. In-depth characterization of the NI, CGA, and CGB. a) Donut plots: inner plot shows the overall contribution (%) of each cluster to the total NI, CGA, and CGB neurons; the outer plot classifies the clusters as glutamatergic (red) and GABAergic (light blue). **b**) Stacked area charts of the NI, CGA, and CGB cluster trajectories*.* Clusters are color-coded according to the legend in panel a. **c**) Right side: MERFISH images show cluster at4_35 NI neurons (cyan polygons) along its marker gene, *Rln3* (magenta spots). Left side: images depicting *Slc32a1*, *Slc17a6*, and *Rln3* transcripts across the same bregma levels. (scale bar:

100 µm). In panel (a), only clusters contributing >0.5 % to the overall neuronal population were plotted.

Supplementary Figure 12. Seurat clustering of all dPnTg human nuclei profiled by snRNA-seq from Siletti et al. dataset a) t-SNE plot of 50,250 nuclei color-coded by annotated cell cluster. **b**) Dot plot of 30 marker genes used to differentiate “neurons” and “glia/non-neuronal cells”. For each cell type, 3 markers were plotted. **c-g**) Stacked bar plots representing the contribution, expressed as % of nuclei (x-axis), of the experimental batch (c) age (d), library construction chemistry (e), dissection strategy (f), and donor (g) covariates to each neuronal cluster (y-axis). All differentially expressed genes in the dot plot have an average log fold-change >0.25 and an adjusted p-value <0.01. Test used: *Wilcoxon Rank Sum two-sided Bonferroni-corrected Test*. t-SNE, t-distributed Stochastic Neighbor. Embedding; OPC, oligodendrocyte progenitor cell; PVM/Micro, perivascular macrophages/ microglia; VSMC, vascular smooth muscle cells; NK, natural killer cell; ECs, endothelial cells NA, no marker detected; CONT, glia contamination.

Supplementary Figure 13. snRNA-seq transcriptional profile of the human dPnTg and cluster correspondence between human snRNA-seq and mouse snRNA-seq datasets. a) t-SNE plot of 50,250 nuclei. **b**) Donut plot depicting each cell type's fraction (%) identified. Color-coded legend for panels a and b is in panel b. **c-d**) t-SNE plots of 17,995 neuronal nuclei from the "excitatory" group (c) and 11,871 neuronal nuclei from the "inhibitory" group (d) color-coded by cell cluster according to legends in panels f and g, respectively. **e**) Dot plot of 30 cell markers (y-axis) that univocally identify each cell type (x-axis). For each cell type, 3 markers were plotted. **f-g**) Dot plots displaying the expression level of the top marker gene for the "excitatory" (f) and "inhibitory" (g) neuronal clusters. The top 2 marker genes specify the identity of each cluster. **h**) Heatmap depicting the cluster correspondence between human snRNA-seq and mouse snRNA-seq datasets in the dPnTg. Legend defining the AUROC score and the “match type” (reciprocal vs non-reciprocal) is on the right side of panel h. **i**) Left: stacked bar plot showing the number of clusters with a match (orange) over the total clusters (grey) identified by snRNA-seq in humans and mice. Right: boxplot showing the AUROC scores distribution. The black middle line denotes the median value (50th percentile), while the grey box contains the 25th to 75th percentiles of the dataset. The black whiskers mark the 5th and 95th percentiles, and values beyond these upper and lower bounds, marked with black dots, are considered outliers. All differentially expressed genes in the dot plot have an average log fold-change >0.25 and an adjusted p-value <0.01. Test used: *Wilcoxon Rank Sum two-sided Bonferroni-corrected Test*. t-SNE, t-distributed Stochastic Neighbor Embedding; OPC, oligodendrocyte progenitor cell; PVM/Micro, perivascular macrophages /microglia; VSMC, vascular smooth muscle cell; NK, natural killer cell; ECs, endothelial cells; AUROC, area under the receiver operator characteristic curve, h, *Homo Sapiens*; m, *Mus Musculus* NA, no marker detected; CONT, glia contamination.

Supplementary Figure 14. A subset of GO gene sets moderately contributes to cell-type replicability. a) t-SNE plot of 6,638 neuronal nuclei derived exclusively from human PB dissections. **b)** Dot plots displaying each cluster's top marker gene expression level. **c**) Sankey plot depicting the cluster correspondence between the mouse PB atlas from Pauli et al. and the human PB atlas from Siletti et al. The thickness of the line reflects the AUROC score. **d**) Left: stacked bar plot showing the number of clusters with a match (orange) over the total clusters (grey) identified in human and mouse PB atlases. Right: boxplot showing the AUROC scores distribution. The black middle line denotes the median value (50th percentile), while the grey box contains the 25th to 75th percentiles of the dataset. The black whiskers mark the 5th and 95th percentiles, and values beyond these upper and lower bounds, marked with black dots, are considered outliers. **e-f**) Dot plot depicting two examples of GO hits related to

“neurotransmitters” and “neuropeptides” classes. The size of the dots indicates the number of cells expressing a gene in a cell type. The color reflects the average expression in z-score. The gene expression is first averaged and scaled in each of the two datasets (human and mouse), and then a final value is obtained by averaging across datasets. In both panels, matches are defined by a joint label that uses one of the two top marker genes from human clusters NA, no marker detected; CONT, glia contamination.
