# Supplementary material for: A spatially-resolved transcriptional atlas of the murine dorsal pons at single-cell resolution
Source: Nat Commun. 2024 Mar 4;15:1966. doi: 10.1038/s41467-024-45907-7 (PMC10912765; doi:10.1038/s41467-024-45907-7)
Supplement: Supplementary file 3 — Description of Additional Supplementary Information [file 41467_2024_45907_MOESM3_ESM.pdf]

SUPPLEMENTARY DATA LEGENDS:

Supplementary Data_1. DE analysis related to "all nuclei" from the snRNA-seq mouse dataset of the dPnTg. Test used: *Wilcoxon Rank Sum two-sided Bonferroni-corrected Test*.
Supplementary Data_2. DE analysis related to "excitatory" neurons from the snRNA-seq mouse dataset of the dPnTg. Test used: *Wilcoxon Rank Sum two-sided Bonferroni-corrected Test*.
Supplementary Data_3. DE analysis related to "inhibitory" neurons from the snRNA-seq mouse dataset of the dPnTg. Test used: *Wilcoxon Rank Sum two-sided Bonferroni-corrected Test*.
Supplementary Data_4. MERFISH gene panel. Data table listing the 315 target genes assessed in MERFISH assay. Reported in the table is the following information: gene name, the type of cell targeted (neuron or glia/ non-neuronal cells), the FPKM from mouse brain bulk RNA-seq (see methods), transcript ID, target regions, isoform length, sufficient target regions, gene targetability.
Supplementary Data_5. DE analysis related to "all cells" from the MERFISH mouse dataset of the dPnTg. Test used: *Wilcoxon Rank Sum two-sided Bonferroni-corrected Test*.
Supplementary Data_6. DE analysis related to "excitatory" neurons from the MERFISH mouse dataset of the dPnTg. Test used: *Wilcoxon Rank Sum two-sided Bonferroni-corrected Test*.
Supplementary Data_7. DE analysis related to "inhibitory" neurons from the MERFISH mouse dataset of the dPnTg. Test used: *Wilcoxon Rank Sum two-sided Bonferroni-corrected Test*.
Supplementary Data_8. Spatial localization of "excitatory" neurons from the MERFISH mouse dataset of the dPnTg. Data table reporting the spatial location of the "excitatory" MERFISH clusters according to the Franklin-Paxinos brain atlas. The table shows the following information: percentage of cells per cluster/total cells, number of cells of each cluster, cluster ID, top 5 markers of each cluster, anatomical location of each cluster, and their inclusion in the downstream analyses.
Supplementary Data_9. Spatial localization of "inhibitory" neurons from the MERFISH mouse dataset of the dPnTg. Data table reporting the spatial location of the "inhibitory" MERFISH clusters according to the Franklin-Paxinos brain atlas. The table shows the following information: percentage of cells per cluster/total cells, number of cells of each cluster, cluster ID, top 5 markers of each cluster, anatomical location of each cluster, and their inclusion in the downstream analyses.
Supplementary Data_10. DE analysis related to neurons from the MERFISH atlas 1. Test used: *Wilcoxon Rank Sum two-sided Bonferroni-corrected Test*.
Supplementary Data_11. DE analysis performed between clusters at1_10, at1_11, and at1_6 (as a negative control, *Calca*-) from the MERFISH atlas 1 and cluster at2_2 from the MERFISH atlas 2. Test used: *Wilcoxon Rank Sum two-sided Bonferroni-corrected Test*.
Supplementary Data_12. DE analysis expression related to PB neurons of the Pauli et al. dataset. Test used: *Wilcoxon Rank Sum two-sided Bonferroni-corrected Test*.
Supplementary Data_13. MetaNeighbor output from the comparison of all clusters from MERFISH atlas 2_ plus clusters at1_10 and at1_11 from atlas_1 versus scRNA-seq atlas from Pauli et al. of the mouse PB.
Supplementary Data_14. DE analysis performed between clusters 15 and 16 of the PB's Pauli et al. scRNA-seq atlas. Test used: *Wilcoxon Rank Sum two-sided Bonferroni-corrected Test*.
Supplementary Data_15. DE analysis related to neurons from the MERFISH atlas 2. Test used: *Wilcoxon Rank Sum two-sided Bonferroni-corrected Test*.

Supplementary Data_16. Cell frequency of MERFISH atlas 2 clusters at2_11 (*Skor2*/*Gm47757*) and at2_16 (*Slc6a5*/*Pax2*) estimated across 11 coronal sections, from -4.8 to -5.75 bregma level. In the Excel sheet, there are the cell count and the MERFISH images relative to two gene expression combinations (i.e., *Skor2/Gm47757*, *Slc6a5/Pax2*) across 11 coronal sections from -4.8 to -5.75 bregma levels.

Supplementary Data_17. Data table displaying the anatomical location of each cluster in MERFISH atlas 2 across 10 coronal sections, from -4.95 to -5.7 bregma level. The anatomical locations of the MERFISH atlas 2 clusters have been established according to the Franklin-Paxinos brain atlas. Abbreviations are in Fig 1A.

Supplementary Data_18. Triple RNA scope for *Foxp2*/*Gpr101*/*Pdyn* in the PB complex. In the main Excel sheet, there is the cell count relative to the 7 possible gene expression combinations (i.e., *Foxp2*, *Gpr101*, *Pdyn*, *Foxp2*/*Gpr101*, *Foxp2*/*Pdyn*, *Gpr101*/*Pdyn*, *Foxp2*/*Gpr101*/*Pdyn*) across 5 bregma levels (-4.9, -5.1, -5.3, -5.5, -5.7) for a total two mouse brains. In the Excel sheets named "Supplementary Data 18 A-E" are the cell counts and the images relative to each of the 5 bregma levels for two mouse brains.

Supplementary Data_19. MERFISH detection of *Foxp2*/*Gpr101*/*Pdyn* in the PB complex. In the main Excel sheet, there is the cell count relative to the 7 possible gene expression combinations (i.e., *Foxp2*, *Gpr101*, *Pdyn*, *Foxp2*/*Gpr101*, *Foxp2*/*Pdyn*, *Gpr101*/*Pdyn*, *Foxp2*/*Gpr101*/*Pdyn*) across 5 bregma levels (-4.9, -5.1, -5.3, -5.5, -5.7) for a total two mouse brains. In the Excel sheets named "Supplementary Data 19 A-E" are the cell counts and the images relative to each of the 5 bregma levels for two mouse brains.

Supplementary Data_20. DE related to re-clustering analysis of the MERFISH atlas 2 cluster at2_2 (Calca/Il20ra). Test used: *Wilcoxon Rank Sum two-sided Bonferroni-corrected Test*.

Supplementary Data_21. DE analysis related to the MERFISH atlas 3. Test used: *Wilcoxon Rank Sum two-sided Bonferroni-corrected Test*.

Supplementary Data_22. DE analysis performed between clusters at3_8 and at3_24 of the MERFISH atlas 3. Test used: *Wilcoxon Rank Sum two-sided Bonferroni-corrected Test*.

Supplementary Data_23. DE analysis performed between clusters at3_30 of the MERFISH atlas 3 and at2_5 of the MERFISH atlas 2. Test used: *Wilcoxon Rank Sum two-sided Bonferroni-corrected Test*.

Supplementary Data_24. DE related to re-clustering analysis of the MERFISH atlas 3 cluster at3_0 (Th/Slc18a2). Test used: *Wilcoxon Rank Sum two-sided Bonferroni-corrected Test*.

Supplementary Data_25. DE analysis related to the MERFISH atlas 4. Test used: *Wilcoxon Rank Sum two-sided Bonferroni-corrected Test*.

Supplementary Data_26. MetaNeighbor output from the comparison of MERFISH atlases 1-4 versus snRNA-seq dataset of the mouse dPnTg.

Supplementary Data_27. DE analysis related to "all nuclei" from the snRNA-seq human dataset of the dPnTg from Siletti et al. Test used: *Wilcoxon Rank Sum two-sided Bonferroni-corrected Test*.

Supplementary Data_28. DE analysis related to "excitatory" neurons from the snRNA-seq human dataset of the dPnTg from Siletti et al. Test used: *Wilcoxon Rank Sum two-sided Bonferroni-corrected Test*.

Supplementary Data_29. DE analysis related to "inhibitory" neurons from the snRNA-seq human dataset of the dPnTg from Siletti et al. Test used: *Wilcoxon Rank Sum two-sided Bonferroni-corrected Test*.

Supplementary Data_30. MetaNeighbor output from the comparison of the human dPnTg (snRNA-seq from Siletti et al.) versus the mouse PnTg (snRNA-seq).

Supplementary Data_31. MetaNeighbor GO analysis performed only on "reciprocal" matches from the output of Supplementary Data 30.

Supplementary Data_32. DE analysis related to neurons from only anatomical dissection of the human PB from Siletti et al. Test used: *Wilcoxon Rank Sum two-sided Bonferroni-corrected Test*.

Supplementary Data_33. MetaNeighbor output from the comparison of the human PB dataset (snRNA-seq from Siletti et al.) versus the mouse PB dataset (scRNA-seq from Pauli et al.)

Supplementary Data_34. Cartesian coordinates (x, y) define an ROI's boundaries that contain the entire dPnTg.

Supplementary Data_35. Data table including the average expression reported as FPKM for all gene isoforms detected in a whole mouse brain bulk RNA-seq dataset.

Supplementary Data_36. Data table containing the KEY ID used to correspond to the gene isoform used to design the MERFISH probe-set to the one in the whole mouse brain bulk RNA-seq dataset.
