# Supplementary material for: A spatially-resolved transcriptional atlas of the murine dorsal pons at single-cell resolution
Source: Nat Commun. 2024 Mar 4;15:1966. doi: 10.1038/s41467-024-45907-7 (PMC10912765; doi:10.1038/s41467-024-45907-7)
Supplement: Supplementary file 5 — Reporting Summary [file 41467_2024_45907_MOESM5_ESM.pdf]

nature portfolio

Corresponding author(s): Bradford B. Lowell
Clifford B. Saper
Anne M.J. Verstegen

Last updated by author(s): 12/28/2023

Reporting Summary

Nature Portfolio wishes to improve the reproducibility of the work that we publish. This form provides structure for consistency and transparency in reporting. For further information on Nature Portfolio policies, see our Editorial Policies and the Editorial Policy Checklist.

Statistics

For all statistical analyses, confirm that the following items are present in the figure legend, table legend, main text, or Methods section.

n/a	Confirmed	
☐	☒	The exact sample size (*n*) for each experimental group/condition, given as a discrete number and unit of measurement
☐	☒	A statement on whether measurements were taken from distinct samples or whether the same sample was measured repeatedly
☐	☒	The statistical test(s) used AND whether they are one- or two-sided<br>*Only common tests should be described solely by name; describe more complex techniques in the Methods section.*
☐	☒	A description of all covariates tested
☐	☒	A description of any assumptions or corrections, such as tests of normality and adjustment for multiple comparisons
☐	☒	A full description of the statistical parameters including central tendency (e.g. means) or other basic estimates (e.g. regression coefficient) AND variation (e.g. standard deviation) or associated estimates of uncertainty (e.g. confidence intervals)
☐	☒	For null hypothesis testing, the test statistic (e.g. *F*, *t*, *r*) with confidence intervals, effect sizes, degrees of freedom and *P* value noted<br>*Give P values as exact values whenever suitable.*
☒	☐	For Bayesian analysis, information on the choice of priors and Markov chain Monte Carlo settings
☒	☐	For hierarchical and complex designs, identification of the appropriate level for tests and full reporting of outcomes
☒	☐	Estimates of effect sizes (e.g. Cohen's *d*, Pearson's *r*), indicating how they were calculated

Our web collection on statistics for biologists contains articles on many of the points above.

Software and code

Policy information about availability of computer code

Data collection	-- for MERFISH assay the image acquisition has been done using a proprietary Vizgen software that is included in the MERSCOPE (the name of the machine that performs MERFISH).<br>-- for DroNc-seq assay the data collection has been done as follow: raw sequencing reads were demultiplexed to FASTQ format files using bcl2fastq (Illumina; version 2.20.0). Digital expression matrices (DGE) were generated using the Drop-Seq tools pipeline (https:// github.com/ broadinstitute/Drop-seq, version 2.4.0) as described in detail in the "Method" section.

Data analysis	After image acquisition using MERSCOPE instrument (Vizgen), MERFISH data were analyzed using the MERlin pipeline through Vizgen's MERSCOPE Analysis Computer by selecting the watershed cell segmentation algorithm. Downstream analysis of snRNA-seq and MERFISH were carried out using R (v . 4 . 2 . 3) and Python (v. 3.8). We used functions that belongs to the following R packages: Seurat v.3.2.3, Harmony v.1.0, GIOTTO vl.1.2, Meta Neighbor v 1.14.0.<br>Graphics were generated using R (v. 4.2.3) base functions or R packages. Bar plots, scatter plots, box plots, donut plots, staked area charts, line charts, and correlation matrix heat-maps were generated with R base functions or the ggplot2 (v. 3.4.3) and plotly_ (v. 4.10.2), cowplot (v.1.1.1), corrplot (v. 0.92), streamgraph (v. 0.9.0) packages. Sankey plots were generated with the networkD3 package (v. 0.4). Dot plots, t-SNE, and violin plots were generated using functions built in the Seurat v3.2.3 package. Voronoi plots were generated using the functions built in the GIOTTO vl.1.2 package. Human-mouse dot plots were generated using the function built in the MetaNeighbor v1.14.0 package.<br>The schematic in Fig 1a was created with BioRender.com. The schematic in Fig. 2a was created using Adobe Illustrator. The schematics in Fig. 7e and 7h were created using Inkscape.<br>MERlin pipeline used to process MERFISH raw data is available on Zenodo: 10.5281/zenodo.3758540.<br>R and Python code used to generate results in the manuscript are available on Zenodo: https://zenodo.org/records/10103722? and 10.5281/zenodo.10396868.

For manuscripts utilizing custom algorithms or software that are central to the research but not yet described in published literature, software must be made available to editors and reviewers. We strongly encourage code deposition in a community repository (e.g. GitHub). See the Nature Portfolio guidelines for submitting code & software for further information.

Data

Policy information about availability of data

All manuscripts must include a data availability statement. This statement should provide the following information, where applicable:

- Accession codes, unique identifiers, or web links for publicly available datasets
- A description of any restrictions on data availability
- For clinical datasets or third party data, please ensure that the statement adheres to our policy

-- The mouse DroNc-seq raw and processed data generated in this study have been deposited in the GEO database under accession code GSE226809 (https://www.ncbi.nlm.nih.gov/geo/query/acc.cgi?acc=GSE226809).
-- The mouse snRNA-seq raw and processed data from Allen Brain Institute (ref. 25,26) used in this study are available in the Allen Brain Atlas database https://knowledge.brainmap.
org/data/LVDBJAW8BI5YSS1QUBG/collections.
-- The mouse MERFISH raw and processed data generated in this study have been deposited in the Beth Israel Deaconess Medical Center database: https://research.bidmc.harvard.edu/datashare/DataShareInfo.ASP?Submit=Display&ID=7.
-- The mouse PB scRNA-seq raw and processed data from Pauli et al. (ref. 38) used in this study are available in the GEO database under accession code GSE207708 (https://
www.ncbi.nlm.nih.gov/geo/query/acc.cgi?acc=GSE207708).
-- The human snRNA-seq raw and processed data from Siletti et al. (ref. 69) used in this study are available in the Google bucket: https://storage.cloud.google.com/linnarsson-labhuman.
-- Source data are provided with this paper.

Research involving human participants, their data, or biological material

Policy information about studies with human participants or human data. See also policy information about sex, gender (identity/presentation), and sexual orientation and race, ethnicity and racism.

Reporting on sex and gender	N/A
Reporting on race, ethnicity, or other socially relevant groupings	N/A
Population characteristics	N/A
Recruitment	N/A
Ethics oversight	N/A

Note that full information on the approval of the study protocol must also be provided in the manuscript.

Field-specific reporting

Please select the one below that is the best fit for your research. If you are not sure, read the appropriate sections before making your selection.

☒ Life sciences ☐ Behavioural & social sciences ☐ Ecological, evolutionary & environmental sciences

For a reference copy of the document with all sections, see nature.com/documents/nr-reporting-summary-flat.pdf

Life sciences study design

All studies must disclose on these points even when the disclosure is negative.

Sample size	No statistical method was used to predetermine the sample size. However, our sample sizes for MERFISH (685,289 cells) and snRNA-seq (222,592 nuclei) experiments in mice are similar to or bigger than those reported in papers (PMID: 34616063), (PMID: 30385464), (PMID: 34663959) that investigated brain areas of similar extent. Our smallest neuronal clusters includes >150 cells (MERFISH) or >200 nuclei (snRNA-seq).
Data exclusions	--in snRNA-seq (DroNc-seq + 10X), nuclei with 1) mitochondrial gene expression detection rate >10%; 2) hemoglobin gene expression detection rate >5%; 3) <400 or >10,000 unique gene counts, possibly representing empty droplets or cell doublets, respectively, were removed. Cell Bender (PMID: 37550580) was run on the sn-RNAseq data to estimate a threshold of a number of genes per nucleus capable of including only true positive nuclei. Cell Bender suggested discarding nuclei with <400 detected unique gene counts. For the higher cut-off we empirically excluded nuclei with >10,000 unique gene counts by carefully evaluating data distribution (S.Fig. 1b).<br>--in MERFISH, cells with <15 gene counts were removed. The value has been empirically chosen after evaluating the data distribution for the cell gene count. Cells with gene counts <15 represented mostly segmentation artifacts (S.Fig. 6a).<br>--in scRNA-seq from Pauli et al. refer to the method section (PMID: 36317965) for exclusion criteria<br>--in snRNA-seq from Siletti et al., refer to the method section (PMID: 37824663) for exclusion criteria.<br>In all the analyses no biological sample was excluded. All samples passed the QC criteria.
Replication	--in snRNA-seq assay (DroNc-seq +10X from ABA), 39 experimental batches (13 from DroNc-seq and 26 from 1OX v3 from ABA) showed good reproducibility of the cell clustering results. Clustering reproducibility was evaluated by assessing the contribution of each experimental batch (% nuclei) to each neuronal cluster (S.Fig. 2c).<br>--in MERFISH assay, we evaluated 4 mice (2 males and 2 females) for clustering reproducibility. We collected 10 sections per mouse. Clustering reproducibility was evaluated by assessing the contribution of each section (% nuclei) to each neuronal cluster (S.Fig. 7c). Each batch included a thick a section spanning a brain area (approximately 1mm) from bregma level -4.7 to -5.8 of the Franklin-Paxinos mouse brain atlas. We sampled every 80-90 microns a section of 10 microns. The other 3 mice did not contain all the levels and were used for pilot experiments; therefore, they were not included to evaluate cluster reproducibility.
Randomization	No randomization was performed for sample collection and analysis. This was not required since we did not perform any comparison between different conditions or treatments.
Blinding	No blinding was performed for sample collection and analysis. This was not required since we did not perform any comparison between different conditions or treatments.

Reporting for specific materials, systems and methods

We require information from authors about some types of materials, experimental systems and methods used in many studies. Here, indicate whether each material, system or method listed is relevant to your study. If you are not sure if a list item applies to your research, read the appropriate section before selecting a response.

Materials & experimental systems

n/a	Involved in the study
☒	☐ Antibodies
☒	☐ Eukaryotic cell lines
☒	☐ Palaeontology and archaeology
☐	☒ Animals and other organisms
☒	☐ Clinical data
☒	☐ Dual use research of concern
☒	☐ Plants

Methods

n/a	Involved in the study
☒	☐ ChIP-seq
☒	☐ Flow cytometry
☒	☐ MRI-based neuroimaging

Animals and other research organisms

Policy information about studies involving animals; ARRIVE guidelines recommended for reporting animal research, and Sex and Gender in Research

Laboratory animals	C57BL/6J background mice from the Jackson Laboratory (JAX) of age between 8-10 weeks of both sexes were used for MERFISH and DroNc-seq assays. Mice were housed at 25°C, ~ss% humidity, on a 12:12-h light/dark cycle. --PB centered dissections used Ai14 mouse (JAX, stock no. #007914, Gt(ROSA)26Sortm14(CAG-tdTomato)Hze) --Bar cenetered dissections used Crh-lRES-Cre mice (JAX, stock no. #012704, B6(Cg)-Crhtml(cre)Zjh/J) crossed with EGFP-Ll0a (JAX, stock no. #024750, B6;129S4-Gt(ROSA)26Sortm9(EGFP Rpll0a)Amc/J) to obtain Crh-lRES-Cre::EGFP-Ll0a mice.<br><br>OTHER ORGANISMS: A Cre-expressing adeno-associated virus, AAVl-hSyn-Cre (pENN-AAVl-hSyn-Cre-WPRE-h GH; titer ≥ lxl013 vg/mL; Addgene, 105553), was injected into the NTS of an Ai14 mouse

Wild animals	No wild animals were caught or obtained.
Reporting on sex	--in DroNc-seq A total of 9 and 8 batches (3-5 mice each) of male and female C57BL/6J mice from JAX, respectively were used.<br>--in MERFISH A total of 7 C57BL/6J mice (4 males and 3 females) 8-10 week-old from JAX were used.
Field-collected samples	No filed-collected samples were caught, used or obtained
Ethics oversight	Mice were treated in accordance with guidelines from the National Institute of Health Guide for the Care and Use of Laboratory Animals. All protocols were approved by Beth Israel Deaconess Medical Center Institutional Animal Care and Use Committee and all efforts were directed to minimize the number of animals and their suffering. Animal experiments were approved by the Beth Israel Deaconess Medical Center's Institutional Animal Care and Use Committee (IACUC) (protocol no. 047-2022).

Note that full information on the approval of the study protocol must also be provided in the manuscript.

Plants

Seed stocks	N/A
Novel plant genotypes	N/A
Authentication	N/A
